# An interpretable MRI-based radiomics model predicting the prognosis of high-intensity focused ultrasound ablation of uterine fibroids

**DOI:** 10.1186/s13244-023-01445-2

**Published:** 2023-07-19

**Authors:** Chengwei Li, Zhimin He, Fajin Lv, Yang Liu, Yan Hu, Jian Zhang, Hui Liu, Si Ma, Zhibo Xiao

**Affiliations:** 1 State Key Laboratory of Ultrasound in Medicine and Engineering, College of Biomedical Engineering, Chongqing Medical University, Chongqing, China; 2 Department of Radiology, The First Affiliated Hospital of Chongqing Medical University, Chongqing, 400016 China

**Keywords:** Machine learning, Radiomics, HIFU, Uterine fibroid, Magnetic resonance imaging

## Abstract

**Background:**

Accurate preoperative assessment of the efficacy of high-intensity focused ultrasound (HIFU) ablation for uterine fibroids is essential for good treatment results. The aim of this study was to develop robust radiomics models for predicting the prognosis of HIFU-treated uterine fibroids and to explain the internal predictive process of the model using Shapley additive explanations (SHAP).

**Methods:**

This retrospective study included 300 patients with uterine fibroids who received HIFU and were classified as having a favorable or unfavorable prognosis based on the postoperative nonperfusion volume ratio. Patients were divided into a training set (*N* = 240) and a test set (*N* = 60). The 1295 radiomics features were extracted from T2-weighted imaging (T2WI) and contrast-enhanced T1-weighted imaging (CE-T1WI) scans. After data preprocessing and feature filtering, radiomics models were constructed by extreme gradient boosting and light gradient boosting machine (LightGBM), and the optimal performance was obtained by Bayesian optimization. Finally, the SHAP approach was used to explain the internal prediction process.

**Results:**

The models constructed using LightGBM had the best performance, and the AUCs of the T2WI and CE-T1WI models were 87.2 (95% CI = 87.1–87.5) and 84.8 (95% CI = 84.6–85.7), respectively. The use of SHAP technology can help physicians understand the impact of radiomic features on the predicted outcomes of the model from a global and individual perspective.

**Conclusion:**

Multiparametric radiomic models have shown their robustness in predicting HIFU prognosis. Radiomic features can be a potential source of biomarkers to support preoperative assessment of HIFU treatment and improve the understanding of uterine fibroid heterogeneity.

**Clinical relevance statement:**

An interpretable radiomics model can help clinicians to effectively predict the prognosis of HIFU treatment for uterine fibroids. The heterogeneity of fibroids can be characterized by various radiomics features and the application of SHAP can be used to visually explain the prediction process of radiomics models.

**Graphical Abstract:**

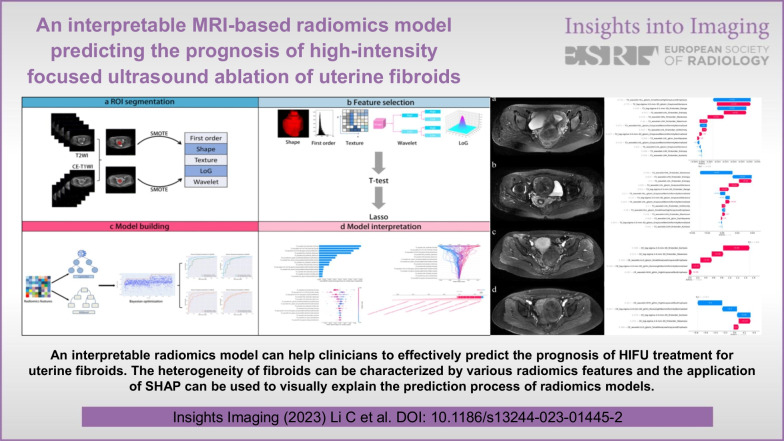

## Key points

- Radiomics models can be used to effectively predict the prognosis of fibroids treated with HIFU.
- The heterogeneity of fibroids can be characterized by various radiomics features.
- The application of SHAP can be used to visually explain the prediction process of radiomics models.

## Background

Uterine fibroids are the most common benign uterine tumors in women of reproductive age [1, 2], but some patients experience abnormal uterine bleeding, pelvic pressure and infertility, which seriously affect their quality of life [3, 4]. High-intensity focused ultrasound (HIFU), a minimally invasive therapy for uterine fibroids, can effectively and safely relieve the patient’s clinical symptoms [5–7]. The non-perfused volume ratio (NPVR) after HIFU ablation is an evaluation criterion for the success of HIFU ablation of uterine fibroids [8]. After long-term follow-up, patients with a larger NPVR had more obvious symptom relief and a lower probability of recurrence and reintervention [9]. However, not all fibroids are suitable for HIFU ablation, and accurate preoperative assessment is important for the efficacy of HIFU treatment.

Magnetic resonance imaging (MRI) is an important tool for the preoperative evaluation and efficacy prediction of HIFU ablation for uterine fibroids [10, 11]. There is an evident link between the variability of uterine fibroid imaging appearance and the heterogeneity of histological presentation [12]. Uterine fibroids are composed of bundles of smooth muscle fibers surrounded by collagenous fibrous connective tissue containing blood vessels. For different types of fibroids, the density of these tissues varies. Due to these differences, uterine fibroids respond differently to HIFU ablation, and fibroids with high signal intensity on T2-weighted imaging (T2WI) and contrast-enhanced T1-weighted imaging (CE-T1WI) scans are considered difficult to ablate successfully [13–17]. However, some researchers have demonstrated positive outcomes using HIFU for fibroids with high signal intensity on MRI [18]. The reason for this is that physicians relying only on subjective evaluation of the signal intensity of fibroids on MRI cannot accurately assess the proportion of tissue components within the fibroids, and that different fibroid separation and targeted fibroid volumes can lead to different ablation results for fibroids of the same signal type. Inaccuracy in MRI assessment undermines the potential benefits of HIFU treatment for uterine fibroid patients. Therefore, additional identification methods and further exploratory studies on the biology of uterine fibroids on MRI are needed.

Radiomics can achieve this goal by extracting and analyzing high-throughput information from conventional grayscale images, transforming the images into quantitative image descriptors related to shape and texture information of the region of interest (ROI) that reflect the characteristics and heterogeneity of fibroids and capture subtle differences that are imperceptible to human vision, and providing physicians with a reference for decision-making [19–21]. Our team has used radiomics to predict the prognosis of HIFU ablation for uterine fibroids [22], but the “black box” nature of machine learning makes it difficult to explain why certain predictions are made for patients. The interpretation of machine learning is inherently a multifaceted concept, for example, what is being interpreted? Who needs interpretability? Why is interpretability needed? To better interpret medical AI systems, the impact of internal features on outcomes needs to be explained for physicians to understand the entire decision-making process so that they can trust the decisions made by the models. However, the medical data used for modeling are often complex, ambiguous and heterogeneous, which makes interpretation extremely challenging [23, 24]. To overcome the “black box” problem, Lundberg and Lee proposed the Shapley additive explanations (SHAP) method to improve the interpretability of a model [25]. A positive or negative value indicates the direction of influence, and the magnitude of the value describes the “weight” or “importance” of the feature. It can help us understand the role of each feature for the overall samples and for individual samples in the prediction process. The combination of SHAP and radiomics illustrates the model in an interpretable way, thereby increasing the credibility of the radiomics model for physicians and patients.

In this study, we aimed to construct MRI-based radiomics models to predict the prognosis of HIFU ablation of uterine fibroids. At the same time, we combined SHAP technology to intuitively explain the decision-making process, understand the relationship between radiomics features and the prognosis of HIFU treatment, i.e., to improve the reliability of the model for physicians and patients.

## Methods

### Study population

This was a single-center retrospective study that was approved by the Ethics Review Committee, and patient consent was abandoned. We enrolled 1055 patients with uterine fibroids who received HIFU treatment from January 2013 to December 2017 and underwent pelvic MRI before and after HIFU treatment. The inclusion criteria were the following: (1) age > 18 years; (2) premenopausal or perimenopausal women; (3) no previous history of relevant surgical or pharmacological treatment; (5) women who were not menstruating; (6) women with an anteverted uterus; (7) fibroid diameter: 3–8 cm; (8) subcutaneous fat thickness: 1–3 cm; and (9) for multiple fibroids, the largest size was included. The exclusion criteria were the following: (1) history of other gynecological conditions, such as endometriosis or pelvic inflammatory disease; (2) pregnancy and lactation; and (3) abdominal scarring.

Previous studies have shown that patients with an NPVR of 80% have obvious symptom relief and a low recurrence rate [26]. Therefore, we defined an NPVR greater than 80% as a favorable prognosis and an NPVR less than 80% as an unfavorable prognosis, and then divided the patients into two groups. Patients were randomly divided into a training set and a test set at a ratio of 8:2.

### MRI data acquisition

In this study, each patient underwent MRI with a 3.0 T system (GE Signa HDxt) before and after HIFU ablation, respectively. The postoperative MRI examination was performed within 7 days after treatment. T2WI was performed using the following parameters: repetition time (TR)/echo time (TE), 270/2.1 ms; field of view (FOV), 98.1 × 38 cm; slice thickness/gap, 6 mm/8 mm; and matrix, 512 × 512; CE-T1WI was performed using the following parameters: repetition time (TR)/echo time (TE), 3.84/1.81 ms; field of view (FOV), 68.4 × 26.5 cm; slice thickness/gap, 4 mm/2 mm; matrix, 512 × 512. The protocol for conventional three-phase-enhanced MRI was to scan arterial phase images at 20 s after injection of GA-DTPA (0.1 mmol/kg, 2.0 mL/s), then scanning the venous phase image 30 s later, and then scanning the delayed phase image 60 s later. MR axial images were exported from PACS and stored in DICOM format for further radiomics feature extraction.

### Tumor masking and radiomics feature extraction

**Fig. 1:**
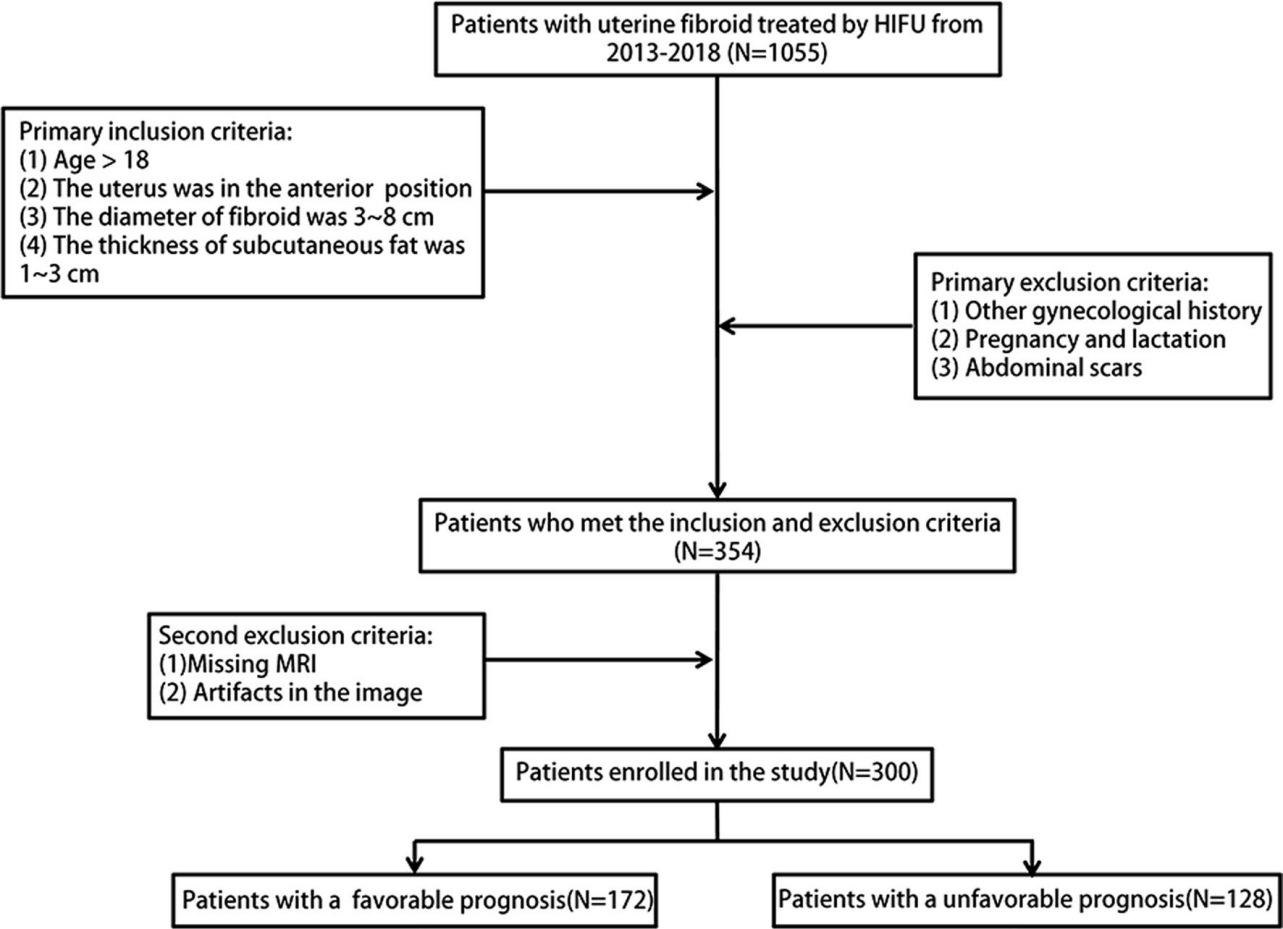
Flowchart of patient enrollment and exclusion

Figure 1 shows the radiomics pipeline of the study. Two radiologists manually outlined the whole fibroids on T2WI and venous phase images of CE-T1WI using ITK-SNAP software, creating an ROI of uterine fibroids. The radiomics package for Python (version 3.7.6) was used to extract radiomics features based on ROI shape and texture for T2WI and CE-T1WI, respectively. We extracted a total of 1295 high-dimensional features and low-dimensional features at this stage. The low-dimensional features included shape features, first-order histogram features, and the high-dimensional features included texture features: gray level co-occurrence matrix (GLCM) features, gray level run length matrix (GLRLM) features, gray level region size matrix (GLSZM) features, neighborhood gray tone difference matrix (NGTDM) features, gray level dependence matrix (GLDM) features, features obtained from the texture matrix in the Gaussian Laplace filtered domain (2.0–5.0 mm kernel), and features from the texture matrix in the wavelet filtered domain.

### Radiomics features reproducibility evaluation

Intraclass correlation coefficients (ICCs) were used to evaluate the consistency and robustness of extracted features from the different ROIs in the same images between two observers. Features with ICC values greater than 0.75 suggested excellent consistency, and features with ICC values less than 0.75 were removed.

### SMOTE balanced data

Data category imbalance may cause the results of the model to be skewed toward the category with more data and reduce the reliability of the model. To address this problem, we used the synthetic minority oversampling technique (SMOTE), which increases the sample of minority categories [27, 28].

### Radiomics feature selection and dimension

Normalization is used to preprocess the data before filling missing values and balancing outliers. The two-sample test (T test) was performed initially to exclude and thereby reduce poor correlation and duplicate features. The final feature selection approach was the least absolute shrinkage and selection operator (LASSO), which was performed to reduce highly correlated features in the selection process and avoid collinearity. The selected features were min–max normalized to accelerate model training and optimize model performance.

### Machine learning models

The radiomics models were constructed by extreme gradient boosting (XGBoost) and light gradient boosting machine (LightGBM) [29, 30]. Bayesian optimization was applied to tune hyperparameters for better model performance [31]. The classification performance was evaluated in the training set and validated in the test set. The predictive performance was quantified by calculating the area under the curve (AUC), accuracy, sensitivity, and specificity. We compared the performance between the four models and selected the model with the highest AUC for further study.

### Model interpretability with SHAP

The SHAP technique was applied to interpret and understand the radiomic features used in the radiomics models. It can be used to visualize the importance of each feature in the overall complex machine learning model and explain how each feature in the model increases or decreases the probability of a single output.

### Statistical analysis

Radiological characteristics of patients with uterine fibroids in the training set and test set were tested for normality using the Kolmogorov‒Smirnov method, and *x* ± *s* was used for data conforming to a normal distribution; *M*(Q25, Q75) was used for data not conforming to a normal distribution. The independent samples T test or Wilcoxon rank sum test was used for quantitative data, and the chi-square test or Fisher’s exact test was used to compare qualitative data. The DeLong test was performed between the models that were constructed using the same sequence features. A *p* value of < 0.05 was used as the level of statistical significance in all statistical tests.

## Results

### Demographic and clinical data

**Fig. 2:**
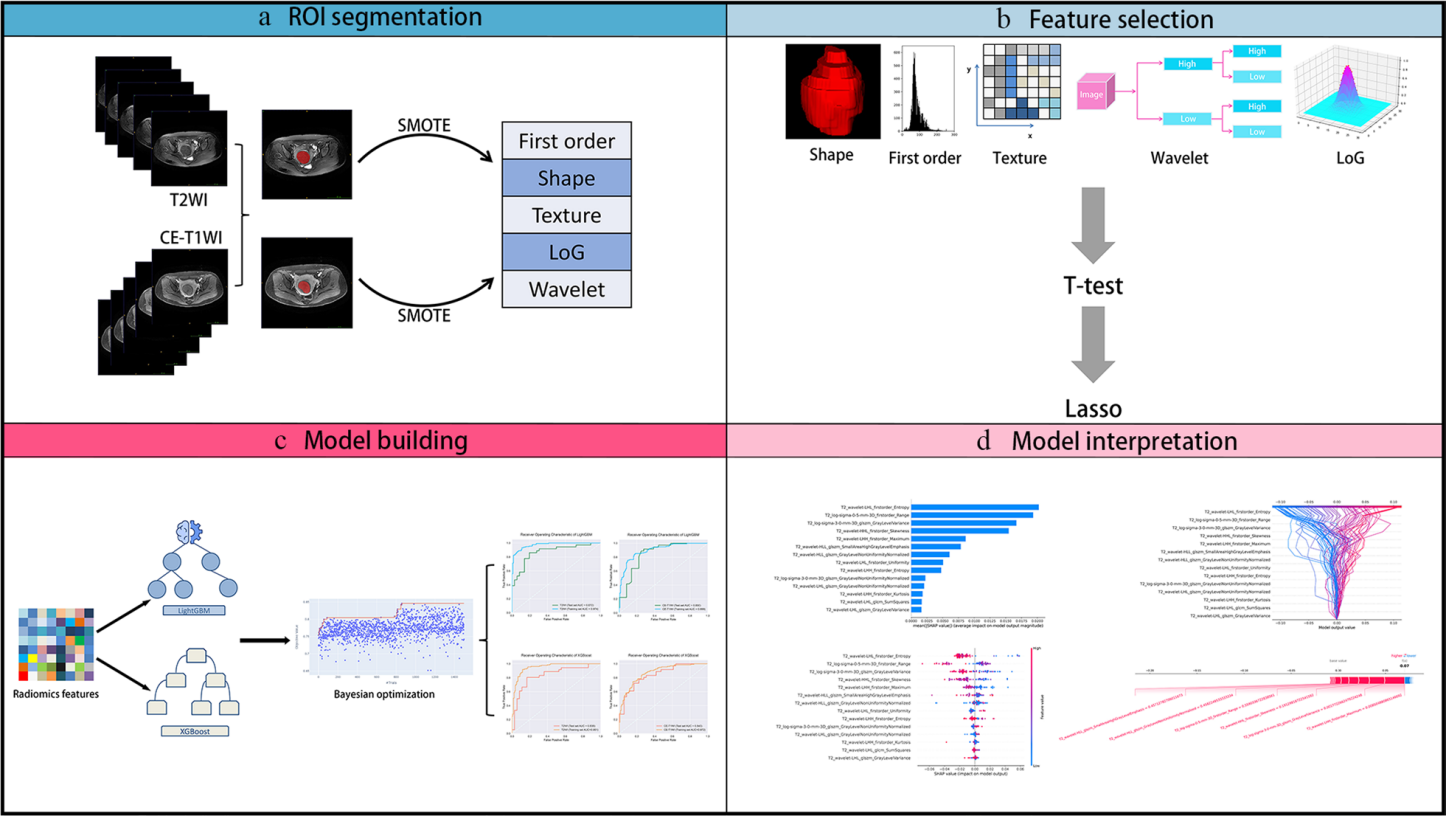
Workflow of the radiomics analysis in this study

Screening was performed according to the inclusion and exclusion criteria, and 354 patients were eligible, including 54 patients with incomplete images and artifacts. Finally, 300 eligible patients were enrolled, namely, 170 patients with solitary uterine fibroids and 130 patients with multiple uterine fibroids. Of these, 128 patients had unfavorable prognoses and 172 patients had favorable prognoses. Additionally, the patients were randomly divided into a training set (*N* = 240) and a test set (*N* = 60) for model construction and validation. Figure 2 shows the flowchart of patient enrollment and exclusion. The clinical and radiological characteristics of the patients and the results of the statistical analysis of the training and test sets are shown in Table 1.

**Table 1:** Comparison of clinical and radiological characteristics of the training and test sets

Characteristics	Training set (*n* = 240)		*p* value	Test set (*n* = 60)		*p* value
	NPVR ≥ 80% (*n* = 136)	NPVR < 80% (*n* = 104)		NPVR ≥ 80% (*n* = 36)	NPVR < 80% (*n* = 24)	
Age (years)	40 (35, 44)	40 (35, 44)	0.804^a^	41.0 ± 6.5	38.4 ± 7.3	0.226^b^
Abdominal fat (mm)	14.6 (12.0, 18.6)	16.1 (12.5, 19.9)	0.052^a^	16.7 ± 6.4	16.4 ± 6.5	0.844^b^
Size (mm)	47.7 (40.5, 56.9)	51.6 (46.7, 61.3)	0.001^a^	52.5 ± 10.7	51.1 ± 9.5	0.606^b^
Volume (cm^3^)	55.93 (33.60, 95.71)	71.65 (52.84, 118.59)	0.001^a^	68.99 (46.19, 114.30)	71.71 (43.54, 102.96)	0.928^a^
Type			< 0.001^c^ 0.001c			0.027^c^
Submucosal	3	3		0	0	
Intramural	116	59		32	16	
Subserosal	17	42		4	9	
T2 signal intensity			< 0.001c			0.067^c^
Low intensity	74	55		15	10	
Intermediate intensity	27	17		4	8	
High intensity	35	32		17	6	
T2 signal homogeneity			< 0.001c			0.457^c^
Homogeneous	99	57		19	15	
Inhomogeneous	37	47		17	9	
CE signal homogeneity			0.001^c^			0.357^c^
Less than myometrium	67	55		21	15	
Equivalent to myometrium	51	20		11	4	
Greater than myometrium	18	29		4	5	
CE signal homogeneity			0.870^c^			0.285^c^
Homogeneous	47	37		13	12	
Inhomogeneous	89	67		23	12	

^a^*p* values were obtained by using Wilcoxon rank sum test; ^b^*p* values were obtained by using independent sample t test; ^c^*p* values were obtained by using the Chi-squared test

### Pre-modeling data preparation

A total of 1295 features were extracted from T2WI and CE-T1WI scans, including the features of 7 radiomics clusters. After ICC consistency analysis, the ICC value of 1 feature in T2WI was less than 0.75, and the ICC value of four features in CE-T1WI was less than 0.75, and they were eliminated. To exclude the impact of data imbalance on the final model performance and interpretability, SMOTE was applied for data balancing, and 44 new patients with NPVR of less than 80% were generated and then added for the follow-up study. Finally, data downscaling was performed using the t test, with 163 features retained in T2WI and eight features retained in CE-T1WI. LASSO was then performed to remove features with little impact on the classification task, and 14 and 5 features were retained in T2WI and CE-T1WI, respectively.

### Performance of the radiomics models

**Fig. 3:**
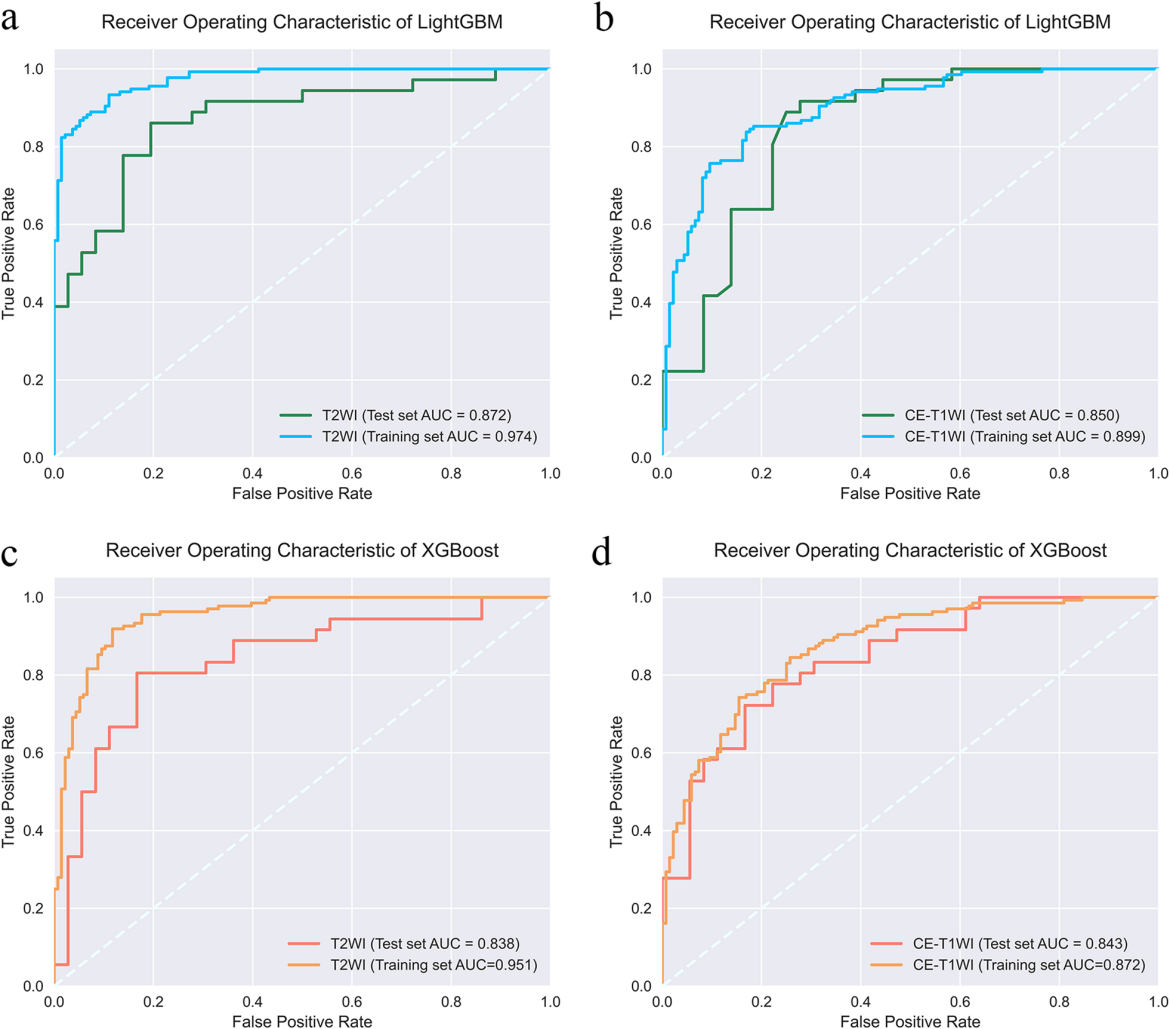
Comparing the AUC of different models. **a** T2WI and (**b**) CE-T1WI models constructed by LightGBM. **c** T2WI and (**d**) CE-T1WI models constructed by XGBoost

The selected features of T2WI and CE-T1WI were used for model construction by LightGBM and XGBoost, and four models with better performance were generated by Bayesian optimization. The LightGBM algorithm generated the best performance for the T2WI and CE-T1WI models with AUCs of 87.2 (95% CI = 87.1–87.5) and 84.8 (95% CI = 84.6–85.7), respectively, while the XGB algorithm obtained models with AUCs of 83.8 (95% CI = 82.9–84.2) and 84.3 (95% CI = 84.1–84.9). Figure 3 shows a comparison of the AUCs of the four models. The rest of the model performance comparisons are summarized in Table 2.

**Table 2:** Performance comparison of LightGBM models and XGBoost models

Comparison	Predictive models		AUC [95% CI]	Accuracy	Precision	Sensitivity	Specificity	*p* value
LGB	T2WI	Training set	0.974 [0.973–0.974]	0.897	0.903	0.890	0.891	
		Test set	0.872 [0.871–0.875]	0.806	0.75	0.917	0.893	0.023
	CE-T1WI	Training set	0.899 [0.894–0.899]	0.831	0.821	0.846	0.841	
		Test set	0.848 [0.846–0.857]	0.750	0.821	0.639	0.705	0.030
XGB	T2WI	Training set	0.951 [0.947–0.955]	0.886	0.852	0.934	0.927	
		Test set	0.838 [0.829–0.842]	0.750	0.701	0.861	0.82	0.023
	CE-T1WI	Training set	0.872 [0.864–0.873]	0.783	0.789	0.772	0.777	
		Test set	0.843 [0.841–0.849]	0.750	0.846	0.610	0.700	0.030

*p* values were obtained by performing DeLong test between LightGBM and XGBoost models constructed using the same features

### Model interpretability with SHAP

**Fig. 4:**
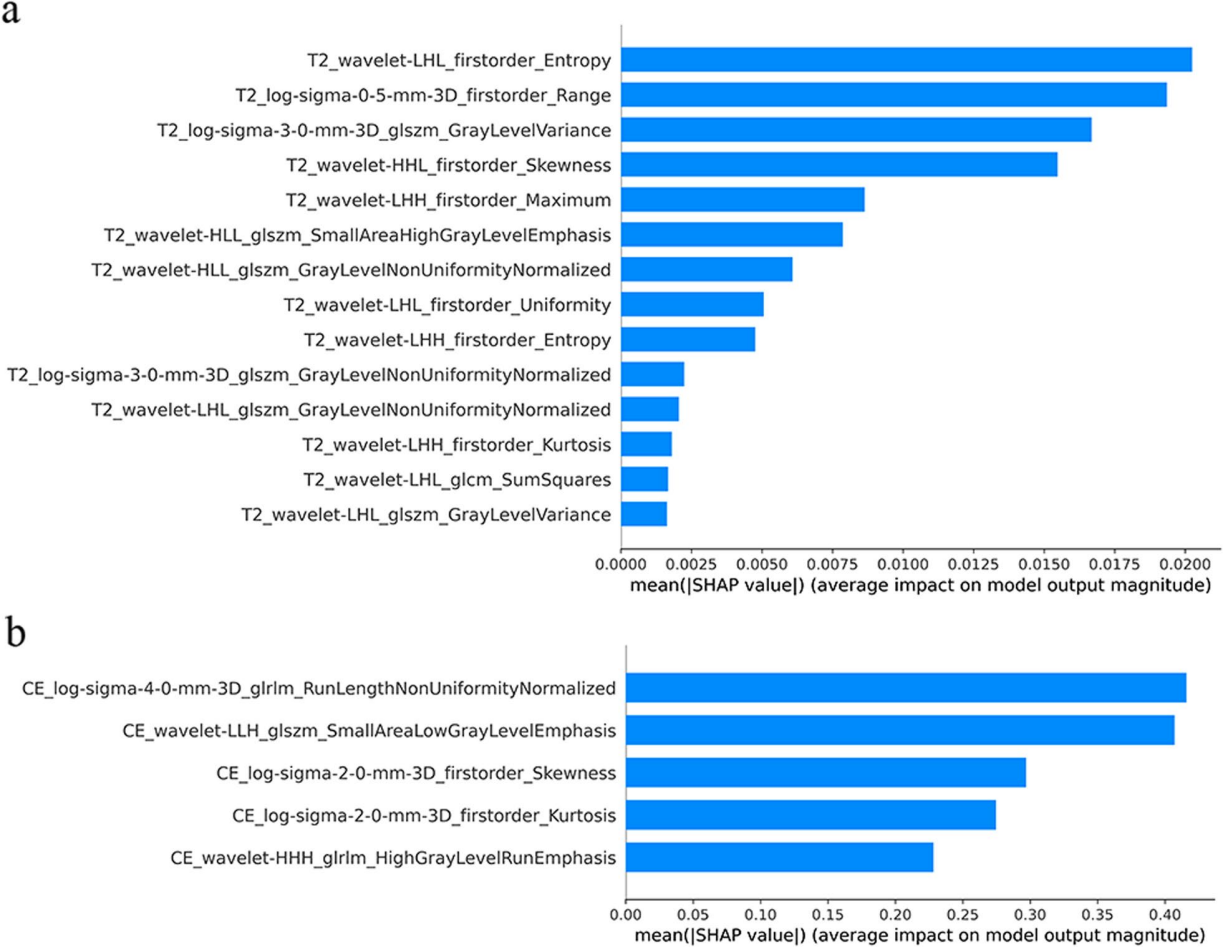
The SHAP feature importance plots of **a** T2WI and (**b**) CE-T1WI models. The plot illustrated the importance of each feature for the global prediction result in descending order

**Fig. 5:**
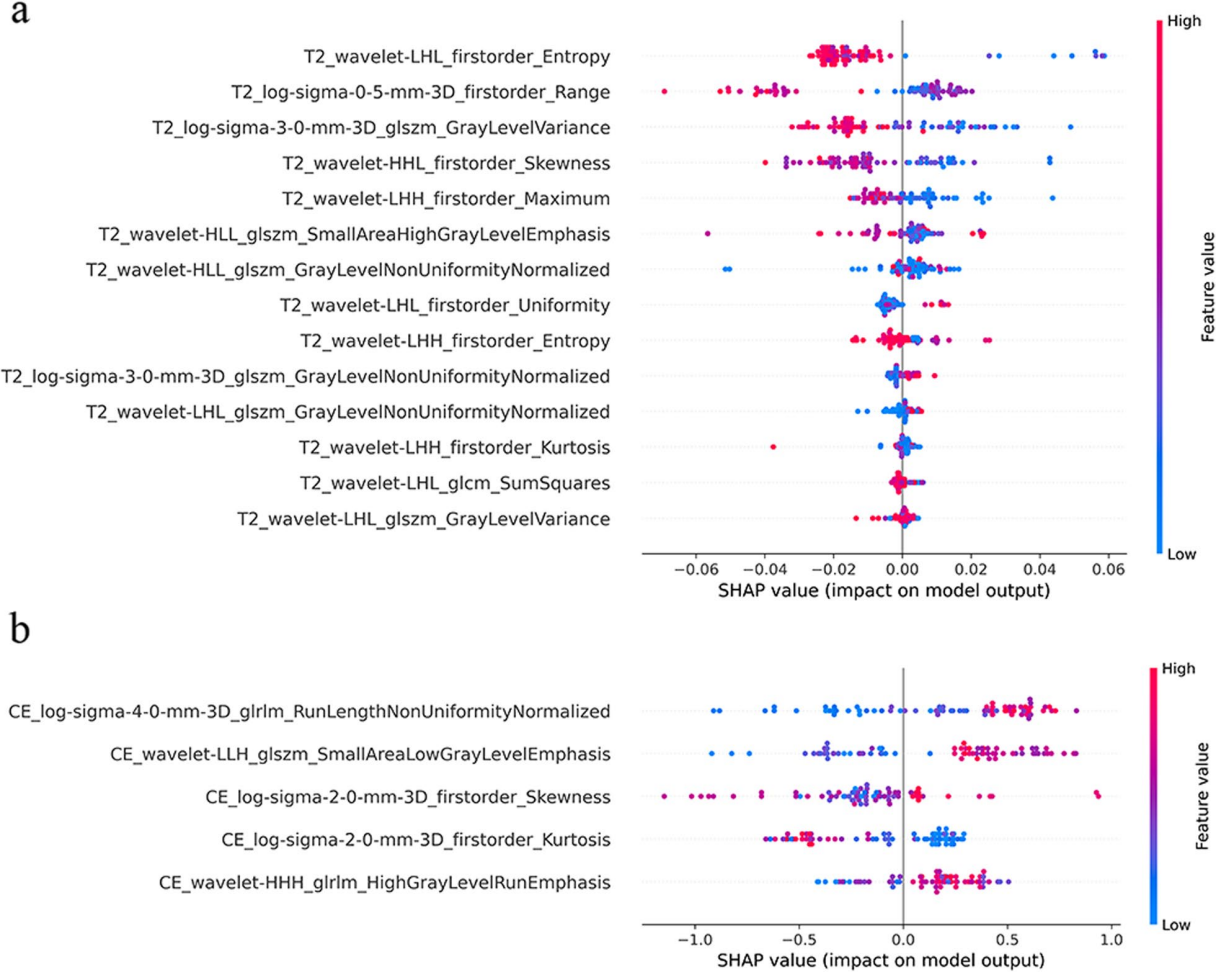
The SHAP summary plot depicted features of global prediction impact on the decision and interaction between features. The importance of features was listed top-down. Each point represents the SHAP value of a patient feature. Dots to the left of the Y-axis increase the chances of having a favorable prognosis, while dots to the right decrease the chances

**Fig. 6:**
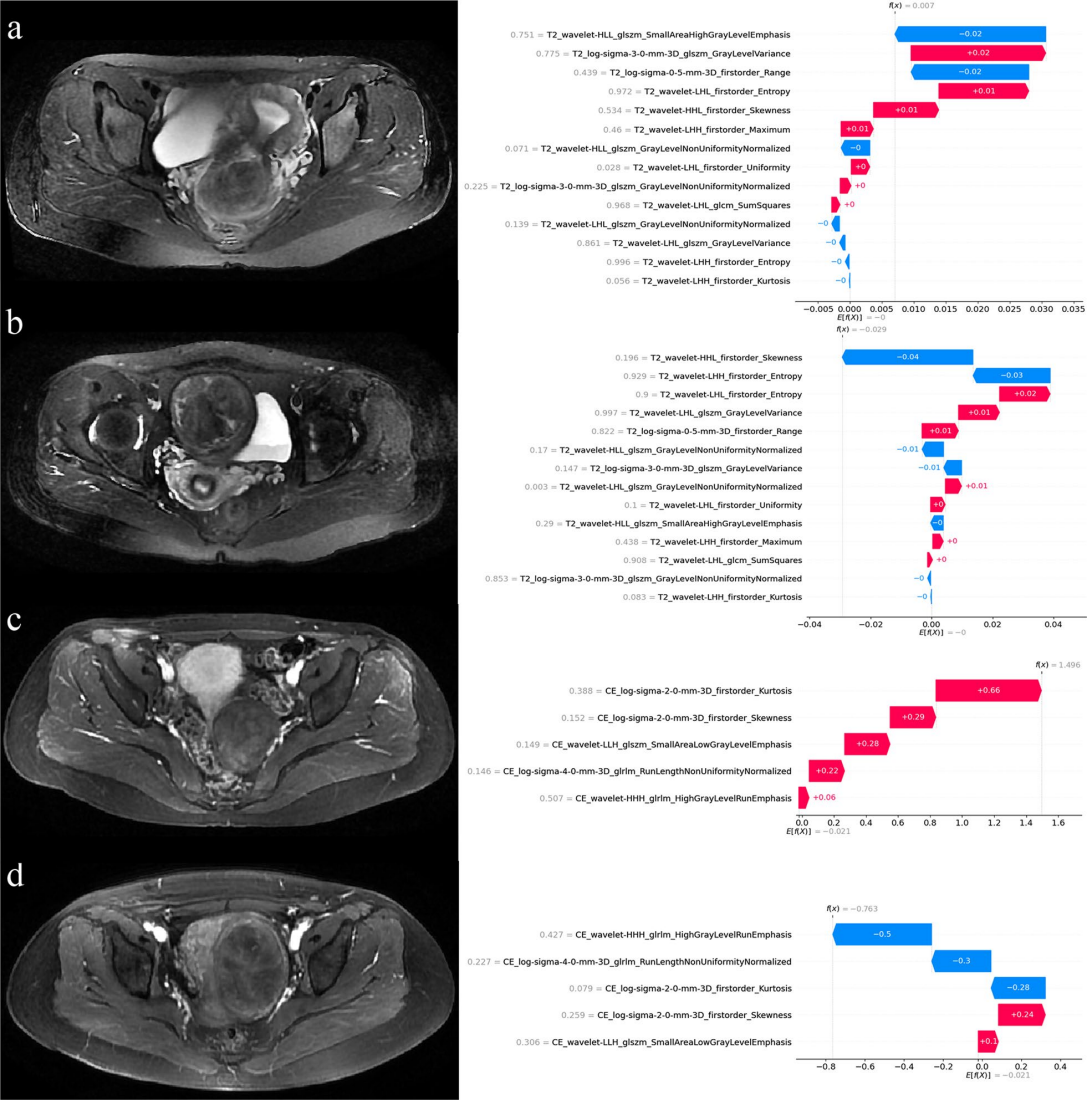
The SHAP waterfall plots showed the individual interpretability of T2WI and CE-T1WI models. Red bar indicates increased predictive value and blue bar indicates decreased predictive value. Under the influence of all features, a final predictive value is obtained, and if this value is less than the base value, the prognosis is predicted to be favorable. Patients (**a**) and (**b**) were randomly selected from the T2WI model as having an unfavorable and a favorable prognosis, respectively. Patients (**c**) and (**d**) were randomly selected from the CE-T1WI model as having an unfavorable and a favorable prognosis, respectively

The SHAP values were calculated for all selected radiomic features included in the best-performing models. Figure 4 shows the SHAP feature importance plot listing the most important features in descending order. The top features contributed more to the model and had higher predictive power than the bottom features. First-order entropy and GLRLM run length nonuniformity normalization were the features of the T2WI and CE-T1WI models that had the strongest impact on the prediction outcomes. As shown in Fig. 5, the SHAP summary plot shows feature impacts on the radiomics model decisions and interactions between features in the model. A positive value of SHAP indicates an increased risk of an unfavorable prognosis for each prediction and vice versa for negative values. The higher the value is, the higher the risk of unfavorable prognosis. In terms of individual sample prediction, we randomly selected four patients from T2WI and CE-T1WI models to make the SHAP waterfall plot (Fig. 6), which depicted the SHAP value of each feature as having a positive or negative contribution to the outcome, and then the final prediction result was obtained.

## Discussion

We have demonstrated the robustness of radiomics models constructed by LightGBM and XGBoost, which were constructed from 14 and 5 significant radiomic features extracted from T2WI and CE-T1WI scans of uterine fibroids, respectively, for predicting the prognosis of uterine fibroids treated with HIFU. These radiomics models, with SMOTE and Bayesian optimization, constructed by LightGBM, showed better performance, with AUCs of 87.2 (95% CI = 87.1–87.5) and 84.8 (95% CI = 84.6–85.7) in the independent test set for the T2WI and CE-T1WI models, respectively, compared to 82.2 for the previously studied model [22]. The model interpretation by the SHAP technique suggested that the radiomics features extracted from the two sequences, which mainly reflected signal intensity, were closely associated with the prognosis of HIFU treatment.

Because multiparametric MRI can show the histological characteristics of uterine fibroids, it is used as an important tool for assessing the efficacy of HIFU for uterine fibroids [32], of which T2WI and CE-T1WI are the most commonly used sequences. The signal intensity on T2WI is attributed to features such as cellularity, vascularity, perfusion, necrosis, edema, and calcification. Conventionally, Funaki types 1 and 2 fibroids are considered good candidates, whereas type 3 fibroids are usually considered poor HIFU responders, although some debulking can be achieved [11, 33]. The signal intensity of CE-T1WI reflects the blood supply of the fibroid, and high signal fibroids are usually difficult to ablate completely [16, 34]. However, the subjective nature of T2WI and CE-T1WI interpretation and the difficulty in distinguishing minor signal intensity changes in tissues are shortcomings of conventional MRI. T2WI and CE-T1WI contain biological information about the cytoarchitectural organization and integrity of fibroids, from which radiomics calculates a large number of quantitative features reflecting microstructural characteristics. We filtered the important features from them to construct efficient supervised learning models. Our results suggested that by using objective and reliable imaging biomarkers, the developed radiomics model has great potential to support evidence-based prediction of the prognosis of HIFU treatment for uterine fibroids in clinical practice. Furthermore, the application of the SHAP technique provides global and local interpretability of radiomics models.

We found that the features retained in the T2WI model were first-order range and first-order maximum, and CE-T1WI model was GLSZM-small area low gray level emphasis, GLRLM-high gray level run emphasis and so on. These features mainly quantify the magnitude of the signal intensity values in the image voxels. The SHAP results demonstrated that larger magnitudes of T2WI and CE-T1WI signal intensities were associated with an increased risk of worse prognosis after HIFU ablation, suggesting that prognosis may be associated with the microscopic tissue and microcirculation that cause hyperintensity in those regions. The main pathological components of uterine fibroids are collagen fibers and smooth muscle cells. The MRI signal of uterine fibroids depends on the ratio of smooth muscle cells and fibrous connective tissue. This means that a higher content of smooth muscle cells and more effective microcirculation of tumor tissue is correlated with worse heat transfer and ablation effects. In contrast, fibrous tissue is more suitable for ultrasound energy deposition, resulting in coagulative necrosis of the target tissue, and leading to better ablation effects. This is consistent with previous studies [35, 36]. In addition, we found that first-order entropy and glrlm run length nonuniformity normalized were the most important features in the two models. These features obtained from the voxel-to-voxel relationship reflect the spatially organized heterogeneity of the fibroids, indicating that the homogeneity of the MRI signal also has a significant impact on the prognosis. High signal fibroids have heterogeneous signals, suggesting a heterogeneous distribution of smooth muscle cells and fibrous tissue in some regions or degeneration such as necrosis and calcification, and ultrasound energy is more easily deposited in this region. Therefore, better ablation results can be achieved with fibroids with heterogeneously high signal intensities [17, 37].

SHAP can be used to provide physicians to explain how the radiomics features of uterine fibroids affect the global prediction results. If the individual prediction process needs to be explained, the SHAP waterfall plot can be used and is considerably faster than the complex scoring system of the difficult nomogram method [38]. It is also clear that the importance of the same features varied between the two groups of cases, and for different patients, features of higher importance may have less impact in some cases. Therefore, SHAP also has good specificity in terms of individual predictions.

There are some limitations in our study. First, this was a retrospective single-center study, and the performance of the models needs to be verified with more multicenter datasets and prospective data. Second, we only selected venous phase images from conventional three-phase scanning CE-T1WI and did not use dynamic-enhanced images, which possibly yielded different results in radiomics analysis and should be investigated in the future. Finally, we did not use diffusion weighted imaging (DWI) for radiomics analysis and interpretation because in previous studies by our group, DWI model performance was worse than T2WI [22], which may be due to its lower image resolution. However, DWI also includes much information about water diffusion and microperfusion in fibroids and needs to be studied using more advanced methods.

## Conclusion

In conclusion, the developed radiomics predictive model, using selected features from T2WI and CE-T1WI sequences, could offer a novel approach to aid clinical assessment of the prognosis of HIFU ablation for uterine fibroids. The SHAP technique can be used to help physicians and patients understand the internal prediction process and increase the credibility of the radiomic models.

## Data Availability

The datasets during the current study are available from the corresponding author upon reasonable request.

## Abbreviations

GLCM: Gray level co-occurrence matrix
GLDM: Gray level dependence matrix
GLRLM: Gray level run length matrix
GLSZM: Gray level region size matrix
HIFU: High-intensity focused ultrasound
LightGBM: Light gradient boosting machine
NGTDM: Neighborhood gray tone difference matrix
NPVR: The non-perfused volume ratio
SHAP: Shapley additive explanations
XGBoost: Extreme gradient boosting
